# Making sense of commercial genomic reports in oncology: a structured approach from clinical practice

**DOI:** 10.3389/fonc.2026.1864818

**Published:** 2026-09-14

**Authors:** Ameer Gharaybeh, Sana Al-Sukhun

**Affiliations:** 1 Stratford Preparatory High School, San Jose, CA, United States; 2 Al Hyatt Oncology Practice, Amman, Jordan

**Keywords:** clinical decision making, genomic databases, molecular tumor board, next-generation sequencing (NGS), precision oncology

## Abstract

**Background:**

Commercial genomic profiling is increasingly used in oncology practice, yet translating reported alterations into treatment decisions remains challenging. Differences across assays, variable evidence levels, and limited access to molecular tumor boards mean that interpretation often depends on the treating clinician rather than the report alone.

**Perspective:**

We describe a structured clinical approach to interpreting commercially generated genomic reports in routine oncology practice using curated public genomic resources, targeted literature review, and clinical feasibility assessment. This approach was developed in a private oncology practice in Jordan to support treatment decisions when formal molecular tumor board access is limited.

**Illustrative application:**

Two clinical scenarios demonstrate both consideration of targeted therapy in the setting of a biologically plausible but weakly annotated alteration and recognition of non-actionability when genomic findings do not justify deviation from disease-specific management.

**Conclusion:**

Structured interpretation can support more consistent clinical decision making around genomic findings, particularly when molecular tumor boards are not readily available. It may help bridge the gap between access to sequencing and meaningful clinical application in routine oncology practice.

## Introduction

Commercial genomic profiling is increasingly used in oncology practice, yet translating reported alterations into treatment decisions remains challenging. Differences across assays, variable levels of clinical evidence, and limited access to molecular tumor boards mean that interpretation often depends on the treating clinician rather than the report alone. The primary challenge in precision oncology is no longer access to sequencing, but the ability to interpret genomic findings in a clinically meaningful way.

Commercial comprehensive genomic profiling (CGP) is often discussed as a single intervention, but in practice it comprises heterogeneous and evolving platforms that differ in gene coverage, sequencing depth, bioinformatic pipelines, and reporting frameworks (1). These differences influence which alterations are detected, how they are classified, and how results are presented for clinical use. Molecular reports are therefore not neutral readouts of tumor biology, but clinical outputs shaped by assay design and interpretation, requiring clinical judgement to determine their relevance for patient management.

Although multidisciplinary molecular tumor boards provide structured interpretation, access remains uneven, particularly in low- and middle-income settings where genomic testing is becoming more available than the expertise needed to interpret it. Public resources such as OncoKB and CIViC provide curated evidence to support genomic interpretation but may lack disease-specific guidance or sufficient data for rare alterations (2–5). As a result, treating clinicians often need to integrate biological plausibility, published evidence, prior therapies, and the feasibility of treatment access when making clinical decisions. In our private oncology practice in Jordan, we developed a structured approach to interpreting commercial genomic reports that combines report triage, review of public genomic resources, targeted literature review, and clinical-feasibility assessment within routine care.

In this report, we illustrate how this approach was applied in two clinical scenarios representing distinct interpretive challenges: a biologically plausible alteration with limited disease-specific evidence, and a genomic profile without a validated actionable target. These cases are presented to illustrate the interpretive process rather than to demonstrate therapeutic efficacy.

## Approach to genomic interpretation

We describe a stepwise structured approach to guide interpretation of commercial molecular profiling reports in routine oncology practice. The approach is based on curated public genomic resources and targeted literature review, without dependence on molecular tumor boards or commercial decision-support systems.

In each case, interpretation was based on a commercially generated molecular report derived from tumor tissue or circulating tumor DNA. Molecular testing was funded through patient self-payment or non-profit support mechanisms.

Molecular profiling was performed by Clinical Laboratory Improvement Amendments (CLIA)-certified and College of American Pathologists (CAP)-accredited reference laboratories. Each patient received a clinical-grade report detailing genomic alterations, associated therapies with levels of evidence, and assay limitations. Molecular reports were not used as stand-alone therapeutic guides but interpreted within clinical context. Each report was evaluated against the structured workflow described below.

### Structured workflow for genomic interpretation

The four-step workflow is summarized in **Figure 1**. The workflow consisted of four steps: (1) report triage, (2) review of public genomic resources (e.g., OncoKB, CIViC), (3) targeted literature search when disease-specific evidence was limited, and (4) clinical feasibility assessment incorporating prior therapy, performance status, drug access, and patient preferences.

**Figure 1:**
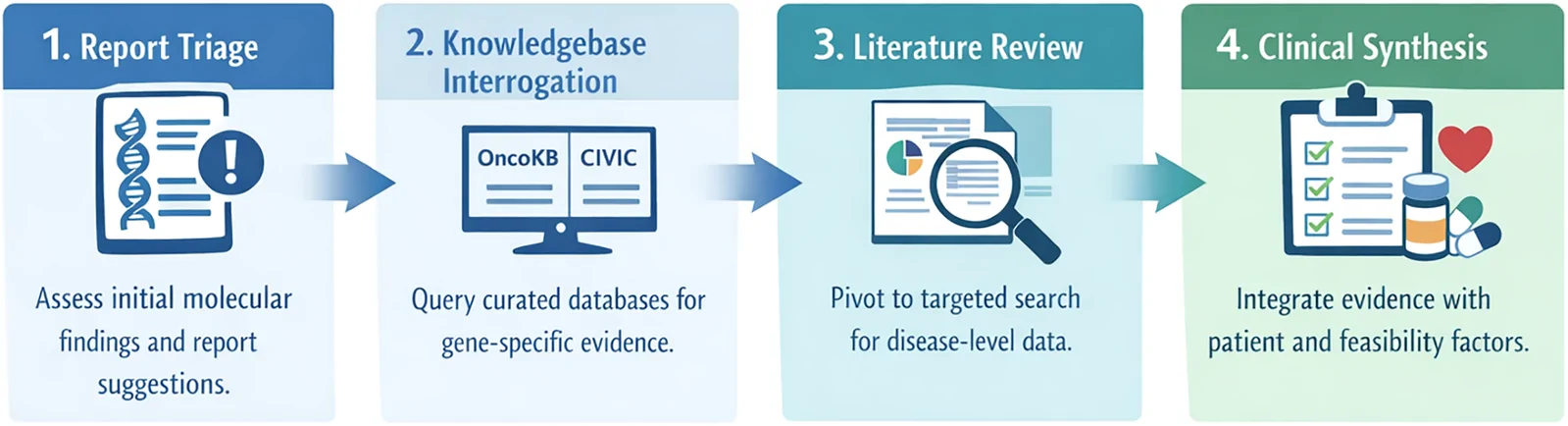
Structured workflow for genomic interpretation.

This retrospective analysis of de-identified data complied with the Declaration of Helsinki. Ethics review was waived, and written informed consent was obtained from both patients.

### Illustrative clinical scenarios

The parallel application of the four-step approach to the two clinical scenarios is summarized in **Table 1**. This structured approach was developed and applied in the course of routine clinical care. The following two cases were selected to illustrate how the approach was applied in practice under two different interpretive conditions: a biologically plausible finding with limited disease-specific evidence, and a profile without a credible actionable target. These cases are presented to demonstrate workflow application rather than therapeutic efficacy.

**Table 1:** Stepwise application of a structured approach to genomic interpretation across two real-world clinical scenarios.

Clinical scenario	Step 1 – Report triage	Step 2 – Knowledgebases	Step 3 – Literature interrogation	Step 4 – Clinical–feasibility synthesis	Outcome
Case 1: (Weak molecular signal) KIT-amplified pancreatic adenocarcinoma	KIT amplification Multiple KIT inhibitors listed No PDAC-specific guidance MSS, no tumor-agnostic marker	OncoKB: likely oncogenic, not actionable in PDAC CIViC: KIT therapies mainly other tumor types	Case reports/small series Cross-tumor KIT dependency	Chemo intolerance Preserved PS Local access to imatinib	off-label KIT inhibition (imatinib)
Case 2 (No actionable driver) Papillary renal cell carcinoma	TP53 truncation FANCD2 indel HGF copy-number gain MSS, low TMB Pazopanib suggested (Level D, gene-linked)	TP53/FANCD2: oncogenic, not actionable HGF gain: MET-axis biology, no validated therapy	Disease-centric search Phase II data in pRCC (pazopanib)	Confirmed papillary histology Good PS Local availability/reimbursement	Histology-guided pazopanib (re-anchored to pRCC evidence)

PDAC, pancreatic ductal adenocarcinoma; pRCC, papillary renal cell carcinoma; MSS, microsatellite stable; TMB, tumor mutational burden; PS, performance status; MTB, molecular tumor board.

This table summarizes the parallel application of the four-step approach to two patients undergoing commercial molecular profiling. Case 1 represents a scenario with a biologically plausible but weakly annotated molecular signal, while Case 2 represents a scenario with no validated actionable genomic driver. For each case, molecular reports were systematically triaged, interrogated using public genomic knowledgebases, supplemented by targeted literature review, and integrated with patient-specific and feasibility considerations to support rational treatment selection.

In both cases, review of public genomic resources alone was insufficient and required escalation to targeted literature review. In one case, a biologically plausible but non-validated alteration supported selection of off-label therapy, whereas in the second, genomic findings did not alter management and treatment was guided by disease-specific evidence. Together, these scenarios illustrate how this structured approach supports both therapeutic decision-making and recognition of non-actionability.

## Case 1: KIT-amplified pancreatic adenocarcinoma with limited disease-specific evidence

A 75-year-old woman with locally advanced pancreatic adenocarcinoma experienced significant toxicity and clinical deterioration, requiring discontinuation of first-line FOLFIRINOX chemotherapy after only two cycles.

### Structured clinical interpretation

#### Step 1: report triage

Circulating tumor DNA profiling (Guardant360^®^) identified high-level *KIT* amplification as the only potentially relevant alteration. The vendor report listed multiple FDA-approved KIT inhibitors, all approved in other tumor types, without pancreatic cancer–specific therapeutic guidance. No guideline-level actionable biomarkers were reported. The tumor was microsatellite stable (MSS) with low tumor mutational burden (TMB). At triage, the report therefore indicated theoretical druggability, but did not establish *KIT* amplification as a validated driver in pancreatic adenocarcinoma or provide a basis to prioritize among proposed agents.

#### Step 2: review of curated public genomic resources

*KIT* amplification was systematically queried in curated public knowledgebases. OncoKB classified the alteration as likely oncogenic, but without standard-care therapeutic associations in pancreatic cancer. CIViC annotated activity of KIT-directed therapies primarily in other tumor types, with no pancreatic-specific therapeutic evidence. This step confirmed biological plausibility but demonstrated that the report’s therapy list lacked disease-associated evidence. Importantly, OncoKB and CIViC did not reach the same conclusion. OncoKB supported the oncogenic relevance of the alteration, whereas CIViC limited therapeutic evidence to other tumor types. Rather than receiving a consistent interpretation, the clinician had to resolve conflicting evidence across knowledge bases. The challenge was further complicated by the fact that, unlike activating KIT mutations, KIT amplification alone does not establish driver status, leaving biological plausibility to clinical judgment rather than the genomic report itself.

#### Step 3: evidence gap analysis and literature interrogation

Because the report’s suggested therapies were anchored to cross-tumor associations rather than pancreatic cancer data, this prompted a focused literature review. A targeted PubMed search (“KIT amplification” OR “CD117” AND “solid tumor” AND “imatinib” OR “KIT inhibitor”) identified case reports and small series describing clinical benefit from KIT inhibition in selected solid tumors, supporting cross-tumor biological rationale while confirming the low level of available evidence.

#### Step 4: clinical–feasibility synthesis

These data were integrated with clinical context, including intolerance to cytotoxic chemotherapy, preserved performance status, and local access to oral KIT inhibitors, they supported selection of imatinib rather than other agents.

#### Therapeutic action & outcome

The patient achieved stable disease with meaningful symptomatic improvement, with a progression-free survival of 5 months before subsequent progression (6).

## Case 2: papillary renal cell carcinoma without a validated actionable genomic driver

A young man presented with metastatic papillary renal cell carcinoma (pRCC), a subtype with limited standard therapeutic options.

### Structured clinical interpretation

#### Step 1: report triage

Tumor NGS (SENTIS™ Cancer+Discovery panel) identified a truncating *TP53* mutation, a *FANCD2* indel, and *HGF* copy-number gain. The tumor was MSS with low tumor mutational burden. The vendor report flagged pazopanib based on low-level, non–disease-specific associations. No guideline-level actionable biomarker was identified.

#### Step 2: review of curated public genomic resources

*TP53*, *FANCD2*, and *HGF* were queried in curated public knowledgebases. OncoKB classified *TP53* as likely oncogenic without therapeutic associations in papillary renal cell carcinoma. CIViC returned no clinically validated therapeutic evidence for *TP53* or *FANCD2*. *HGF* gain suggested MET-axis biology but lacked disease-specific actionable annotation. FANCD2 had no validated therapeutic annotation in pRCC across the major knowledge bases and, in practical terms, was treated as a variant of uncertain clinical significance. Rather than supporting a treatment recommendation, it highlighted the limits of genomic interpretation in this setting. The more difficult challenge involved the vendor’s recommendation of pazopanib based on a gene-linked Level D association. Review of the available knowledge bases did not support that genomic rationale. Pazopanib remained the preferred treatment, but for an entirely different reason: evidence from histology-specific phase II studies in pRCC. This required rejecting the report’s molecular justification while arriving at the same therapeutic choice through disease-specific clinical evidence, a type of judgment that extends beyond what current knowledge bases can provide.This step confirmed biological relevance without a credible gene-directed option.

#### Step 3: evidence gap analysis and literature interrogation

Given the absence of actionable gene-variant evidence, the approach triggered a predefined pivot from gene-centric to disease-centric review. A focused PubMed search identified phase II data demonstrating clinical activity of pazopanib in advanced papillary renal cell carcinoma.

#### Step 4: clinical–feasibility synthesis

The molecular conclusion of non-actionability was integrated with disease-specific evidence, patient factors (good performance status, confirmed papillary histology without clear-cell component), and feasibility considerations, including local availability and reimbursement. This analysis supported use of the report-listed agent on the basis of histology-specific evidence. Pazopanib was therefore recommended as a rational therapy.

#### Therapeutic action & outcome

Treatment with off-label pazopanib was initiated. The patient achieved a durable partial response, which has been maintained for over 36 months with ongoing therapy and minimal toxicity.

## Discussion

This report describes a structured approach to interpreting commercially generated molecular reports in routine oncology practice. The illustrative cases highlight that access to sequencing alone does not guarantee effective precision care; rather, the clinical value of genomic data depends on how they are interpreted. This structured workflow keeps interpretation consistent even when formal molecular tumor board support is limited or unavailable. The purpose of this report is to make the interpretive process explicit rather than to claim therapeutic efficacy. The cases demonstrate how this approach applies across key interpretive scenarios, including situations where evidence is limited and where no actionable target is identified. Importantly, the approach not only supports consideration of targeted therapy but also helps prevent overinterpretation of genomic findings. In the second case, the absence of a validated actionable alteration led to a decision guided by disease-specific evidence, underscoring that structured interpretation is equally valuable in identifying when genomic data should not alter standard management.

The relevance of this approach extends beyond individual cases: in many settings, testing capacity continues to outpace interpretive capacity, particularly in limited-resource environments where molecular tumor boards and precision oncology expertise may not be consistently available. A structured approach based on publicly accessible resources may help address this gap by supporting more consistent and clearer interpretation of genomic findings in routine practice.

Interpretive variability is also influenced by differences in genomic assays, which affect which alterations are detected and how they are reported. Although specific platforms are rarely endorsed by clinical guidelines, commercial assays are developed within established regulatory and professional standards. Consensus recommendations from the Association for Molecular Pathology, ASCO, and the College of American Pathologists provide structured guidance for somatic variant interpretation, including tiered classification, evidentiary annotation, and reporting transparency (7). In parallel, clinical genomic laboratories operate under formal accreditation or regulatory oversight—such as CLIA and CAP in the United States or ISO 15189–aligned frameworks internationally—ensuring baseline standards for analytical validity and reporting quality (8). However, these standards do not eliminate meaningful differences across platforms in genomic scope, assay sensitivity, bioinformatic pipelines, or curation approaches. As a result, the content and interpretation of molecular reports may vary across assays, reinforcing the need for a consistent, clinician-led approach to integrating genomic data into treatment decisions.

The individual components of this workflow, including knowledgebase interrogation and escalation to the published literature, are well established and align with existing practical guidance for NGS report interpretation (9, 10). Both Schmid et al. (9) and Casolino et al. (10) describe these processes within the context of a molecular tumor board and do not address situations in which treating clinicians lack access to such expertise. Our approach differs by making clinical-feasibility synthesis a distinct and explicit final step. It systematically integrates prior therapy, performance status, local drug availability, reimbursement, and patient preferences into treatment selection. In our setting, this assessment is performed by the treating clinician rather than a multidisciplinary committee. Making this decision process explicit, instead of relying on undocumented clinical judgment, is the practical contribution of this report.

This variability in interpretation is reflected in clinical data. Challenges in genomic interpretation are not limited to low-resource settings. Even in well-resourced environments, interpretive concordance remains incomplete. In the ASCO TAPUR molecular tumor board analysis, independent expert panels reviewing identical datasets achieved only partial or full agreement in approximately 79% of cases, reflecting the complexity of variant interpretation and therapeutic prioritization (11). Similarly, fewer than 10% of patients with advanced cancer in the United States ultimately receive genome-directed therapy, highlighting attrition across the precision oncology pathway, including interpretation, trial access, and implementation (12). Data from prospective umbrella and platform trials further show that only a minority of sequenced patients receive matched therapies (13, 14). These findings underscore the need for structured interpretation alongside expanded sequencing capacity. Formal validation of this approach should include concordance studies with molecular tumor boards and prospective evaluation in clinical practice.

Virtual molecular tumor boards (VTBs) have emerged as a practical way to extend molecular interpretation to settings that lack in-house multidisciplinary expertise. For example, an open-access virtual MTB platform in Colombia changed treatment recommendations in just over half of the cases discussed, showing that cross-institutional and cross-border collaboration is both feasible and clinically meaningful in an LMIC setting (15). More broadly, VTBs have been proposed as a way to improve equity in cancer care (16).

Although VTBs help address the need for multidisciplinary interpretation, they do not replace the need for a structured approach at the point of care. This is particularly relevant in settings like ours, where regular access to a VTB is not always available. Rather than serving as alternatives, structured clinician-led interpretation and VTB participation should be viewed as complementary approaches. Assay selection can shape the molecular trajectory of a patient by determining which alterations are detected and reported. In advanced disease, comprehensive profiling is often a one-time opportunity, making interpretation critical. Commercial reports are best considered structured data rather than definitive clinical answers. Within this approach, interpretation is guided by tumor-specific biological plausibility, strength of evidence, and feasibility of therapy access, enabling consistent and clinically grounded decision-making across heterogeneous reports.

The approach accommodates different levels of evidence, from established clinical data to biologically plausible preclinical findings, particularly when evaluating rare alterations or off-label treatment options. It does not replace established evidence frameworks such as ESCAT (17). Instead, it provides a structured way to apply those frameworks within routine clinical decision-making.

Multidisciplinary molecular tumor boards remain the reference standard for genomic interpretation but require substantial resources and are not universally accessible. This approach is intended as a practical adjunct in their absence. Its effectiveness depends on improving genomic literacy among clinicians, as gaps in molecular oncology training persist (18, 19). Integrating precision oncology into medical education, expanding training initiatives, and promoting structured use of public genomic knowledgebases are essential to support consistent, evidence-informed interpretation across diverse clinical settings (9, 20).

## Limitations

This report is intended to illustrate the proposed approach through two purposively selected cases with different interpretive outcomes. It was not designed to determine the applicability of the proposed approach across the broader spectrum of complex real-world clinical scenarios, how frequently it would be applicable in routine practice, whether different clinicians would reach the same conclusions, or whether it improves clinical outcomes compared with standard practice or multidisciplinary molecular tumor board review. Those questions require evaluation in a consecutively sampled cohort, ideally with comparison against molecular tumor board recommendations, and are beyond the scope of this Perspective.

## Data Availability

The original contributions presented in the study are included in the article/supplementary material, further inquiries can be directed to the corresponding author.
